# Geographic Variation in Osteoporotic Hip Fracture Incidence: The Growing Importance of Asian Influences in Coming Decades

**DOI:** 10.4061/2010/757102

**Published:** 2010-08-02

**Authors:** D. K. Dhanwal, C. Cooper, E. M. Dennison

**Affiliations:** ^1^MRC Epidemiology Resource Centre, Southampton General Hospital, University of Southampton, Southampton SO16 6YD, UK; ^2^Botnar Research Centre, Institute of Musculoskeletal Sciences, University of Oxford, Oxford OX3 7LD, UK

## Abstract

Studies over the last few decades have demonstrated geographic variation in the incidence of hip fracture across continents and among different parts of the same region. This paper studies the epidemiology of hip fracture worldwide, with special emphasis on the geographic variation among Asian countries. Using the Pubmed database, keywords that were employed included hip fracture, incidence rate, geographic variation, osteoporosis, and epidemiology. Articles were chosen based on the basis of (1) focus: studies that were said to specifically focus on geographic variation in hip fracture from different continents with a focus on Asia; (2) language: studies that were in English; (3) methods: studies that used statistical tests to examine hip fracture incidence rates. The highest hip fracture rates are seen in Scandinavian countries and the US and the lowest in African countries. Fracture rates are intermediate in Asian populations. Among different ethnic populations, the highest fracture rates are seen in Caucasians and the lowest in blacks. There is also a north-south gradient, particularly in Europe, where more hip fractures occur in North Europe compared to the South.

## 1. Introduction

Osteoporosis is recognised as a major public health problem through its association with low trauma or fragility fracture. Osteoporotic hip fracture is an established health problem in the West over the last six decades and is increasingly being recognised as a growing problem in Asia [1]. With a rising life expectancy throughout the globe, the number of elderly individuals is increasing in every geographical region; the incidence of hip fracture is estimated to rise from 1.66 million in 1990 to 6.26 million in 2050 [2]. All osteoporotic fractures increase patient morbidity; however, the fractures of hip and vertebrae are associated with significant mortality. Hip fracture incidence increases exponentially with age and more so in women. With changing world population dynamics it has been estimated that more than half of these fractures will be seen in Asia by year 2050. Geographic and ethnic variation exists for hip fractures. The exact reasons for this geographic variation are ill understood but genetic factors, less bone mineral content, an aging population and environmental factors such as dietary factors and vitamin D levels are important in the pathogenesis of hip fracture. Understanding this changing epidemiology of hip fracture is therefore essential to develop strategies for the future with a special emphasis on Asia. 

## 2. Methods

This paper was conducted using the Pubmed database. Keywords that were employed included hip fracture, incidence rate, geographic variation, osteoporosis, and epidemiology. The articles were chosen based on the basis of (1) focus: studies that were said to specifically focus on geographic variation in hip fracture from different continents with a focus on Asia; (2) language: studies that were in English; (3) methods: studies that used statistical tests to examine hip fracture incidence rates.

## 3. Review

**Figure 1:**
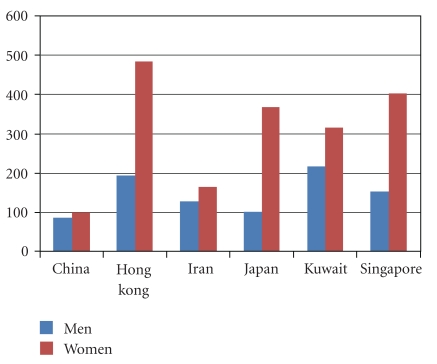
Age-standardized hip fracture rates (per 100,000) across Asian countries.

Age-standardized hip fracture rates (per 100,000) across different continents and Asian countries are given in Table 1 and Figure 1, respectively. The highest hip fractures are seen in US populations but the recent trends show that these fracture rates have either stabilized or decreased in the last two decades. In a population-based study from Minnesota it has been observed that annual age-adjusted incidence rates among women rose rapidly until 1950 only to fall slowly thereafter [3]. Age-adjusted rates in men rose more steadily before beginning a downturn after 1980. Incidence rates rose exponentially with age in both men and women. A Californian study looked at hip fracture rates between 1983 and 2000, with particular interest in the Hispanic population, the largest, fastest growing ethnic minority in the United States [4]. Hip fractures were identified using the annual hospital patient discharge database. Among non-Hispanic white women in California the standardised annual hip fracture rates for those 55 years old and over fell steadily over the past two decades by 0.6% per year in women and 0.5% in men. No significant change occurred among black or Asian women. By contrast annual fracture rates amongst Hispanic women increased by 4.9% per year and 4.2% in men. This supports the hypothesis that residence in early life has a much greater association with variation in hip fracture rates that does current region of residency. Another explanation is that Hispanic men and women have been shown to partake in less physical activity and have a greater risk of nutritional deficits than non-Hispanic whites. In a recently published study by Brauer and coworkers [5], it was concluded that in the US the annual mean number of hip fractures was 957.3 per 100,000 for women and 414.4 per 100,000 for men [5]. The age-adjusted incidence of hip fracture increased from 1986 to 1995 and then steadily declined from 1995 to 2005. The age-adjusted fracture incidence in Canada is 86.4 per 100,000 in women and 53.4 per 100,000 in men [6]. On comparison with data from the US, the overall fracture rate in Canadian women was 30% lower than that in US women in 2001 and 26% lower than that in German women in 2004. Canadian men showed similar overall hip fracture rates to American men prior to age 80 years but 26% lower rate after 80 years. 

In Europe, Scandinavia has the highest reported incidence of hip fracture worldwide. There are a large number of studies looking at the incidence as well as secular trends in this geographically northern region. The incidence rates vary from North to South Europe, the highest being in Sweden and Norway and the lowest in France and Switzerland. From Norway the reported age standardised annual incidence rates of hip fracture are 920 per 100,000 in women and 399.3 per 100,000 in men and those in Switzerland are 346 per 100,000 and 137.8 per 100,000 in women and men, respectively. Studies from Malmo, Sweden showed an exponential increase in hip fracture incidence from 1950 to 1985 in both men and women over age 50, increasing from an annual age-adjusted incidence of 150 to 390 per 100,000 in men and 300 to 830/100,000 in women [7]. A recent study from the UK looked at hospital episode statistics from 1989–1998 [8]. Age-standardised incidence rates increased by 32% in women and 38% in men to 1991-92 and thereafter remained stable. In The Netherlands between 1972 and 1987 the age-adjusted incidence of hip fractures rose linearly from 479/100,000 to 669/100,000 per year in women and from 198/100,000 to 308/100,000 in men aged 65 years and over using the Dutch medical registry [9]. European data on hip fracture shows a clear north-south gradient with the highest hip fractures occurring in Scandinavian countries and the lowest in Spain, and one of the reasons may be that colder winter induces more falls in an elderly population. 

The age-adjusted rates from Australia are 130/100,000 person years in men and 390/100,000 person years in women [10] with no significant change over the last two decades. Women aged 65–75 years were the only age specific group with a 1% decline in annual incidence of hip fracture and the rest being unchanged during the period.

Limited data available from South American countries reveals inconsistent results. This may be due to different methods used for fracture incidence measurement. In a study published from Mexico in 2005, the annual rates of hip fracture in the two public health care systems were 169 in women and 98 in men per 10,000 person years [11]. These fracture rates are similar as reported from southern countries from Europe. Overall the fracture rates are much less than those seen in US and Europe, and the major reason is shorter life span. Only 5.7% of the population lives over 65 years of age. 

A major study concluded that in a Japan population aged 35 years or older the crude incidence of hip fracture was 244.8 per 100,000 person years from 2004 to 2006, and the gender-specific incidence was 99.6 per 100,000 person years for men and 368 per 100,000 person years for women [12]. When data was analysed and compared with that from 30 years ago it was also concluded that there is an increasing incidence of hip fracture in Japanese populations. The highest incidence of hip fracture from Asia has been reported from Singapore. A study by Koh et al. revealed that hip fracture rates from 1991 to 1998 (per 100,000) were 152 in men and 402 in women, and this was 1.5 and 5 times higher than corresponding rates in 1960s [13]. Since 1960 the main increase in hip fracture rates has been seen in Chinese and Malays with rates in Indian ethnic group appearing to decrease. Incidence rates of hip fracture from Hong Kong are 110 per 100,000 in women and 50 per 100,000 in men as per data from public hospitals in 1995 [14]. Secular trends on hip fracture from Hong Kong suggest that over the last three decades the age specific incidence increased 2.5 fold in women and 1.7-fold in men. The incidence rates were found to be similar to those seen in Wessex Health region of UK [15]. In Beijing, China hip fracture incidence was obtained from admissions between 1988 and 1992 within the 76 city hospitals [16]. It was presumed that all the fracture cases from Beijing go to these public hospitals only.

The majority of data from the Middle East is available from Iran from the Iranian Multicenter Study on Accidental Injuries [17]. This study reported age-standardized incidence rates of 127.3 per 100,000 person years in men and 164.6 per 100,000 person years in women which are much lower than those of all the Western countries and US population. There need to be more studies from other parts of Asia especially from India in order to understand the geographic variation in hip fracture in this region as a whole.

Osteoporosis and fragility fractures are believed to be uncommon in Africa. To study this Zebaze et al. conducted a study in Cameroon by documenting all patients aged 35 years and older admitted to the two main urban hospitals in Cameroon following a diagnosis of fracture during two years [18]. Using the 1997 estimates of population, the incidence of low energy trauma fractures (per 100,000 persons over 35 years) was 4.1 in women and 2.2 in men at hip. Similar low fractures rates have been reported from Morocco in 2005 [19]. 

To investigate the geographic variation in different parts of the world and whether this is genuine or related to error in data collection, Schwartz et al. [20] carried out a cross-national study of hip fracture in five geographic areas—Beijing, China; Budapest, Hungary; Hong Kong; Porto Alegre, Brazil; Reykjavik, Iceland—during the years 1990–1992. Cases of hip fracture among women and men of 20 years and older were identified using hospital discharge data in conjunction with medical records, operating room logs, and radiology logs. Estimated rates varied widely, with Beijing reporting the lowest rates (45.4 per 100,000 in men and 39.6) and Reykjavik the highest rates (men = 141.3; women = 274.1). Rates were higher for the women than for the men in all areas except Beijing. The study demonstrated large differences in hip fracture incidence rates, with age-adjusted incidence rates in women being 6 times higher and in men over 3 times higher in Reykjavik compared with Beijing. Results of this study indicate substantial limitation in relying on the hospital discharge data alone to estimate hip fracture incidence rates but the error found in the discharge lists is smaller than the large international variation found. The study concluded that the differences reported among countries mainly reflect genuine variation in the hip fracture incidence rates. 

The influence of ethnicity on risk of osteoporotic fractures was analysed by our group (unpublished). The rates vary considerably according to the geographic area and race and may vary widely within the same country and within populations of a given sex and race. In Europe, hip fracture rates vary 7-fold between countries. In general people who live in latitudes further from the equator seem to have a higher incidence of fracture. The highest rates of hip fracture are seen in Caucasians living in northern Europe, especially Scandinavians. A study from 1989 found that the age-adjusted 1-year cumulative incidence of hip fracture in Norway was 903/100,000 for women and 384/100,000 for men. The rates are intermediate in Asians, China and Kuwait, and the lowest in black populations. While studies in central Norway suggest a stabilisation in fracture rates in recent years, a Californian study published in 2004 reported a doubling of hip fracture rates in Hispanics while no significant change occurred among black or Asian men or women. Many of the lower incidence rates seen in the developing countries can be partially explained by lower life expectancy; in Latin America only 5.7% of the population is over 65. Reduced longevity may also be the explanation for the low fracture rates observed in Morocco.

The reasons for these geographic and ethnic variations are ill understood but factors which may be responsible are genetic factors and environmental factors. Those factors studied so far, such as alcohol consumption, smoking, activity levels, obesity, and migration status, have not explained these trends however. Reduced lifespan in African and Asian population may be one important attribute for the lower incidence of hip fracture in these regions. Further research is clearly needed to explain these important environmental factors. 

## 4. Conclusion

To conclude, osteoporotic hip fractures are responsible for both morbidity and mortality among an elderly population and consume a large amount of health care resources in Europe and the USA. However, a recent decline in the hip fracture incidence in these regions is good news for health authorities but that is not true for the rest of the world. With a changing population profile and increasing elderly population in Asian countries there will be a shift of focus from Europe and US to Asia; health authorities need to prepare to face this challenge in the next four decades.

## Figures and Tables

**Figure 2:**
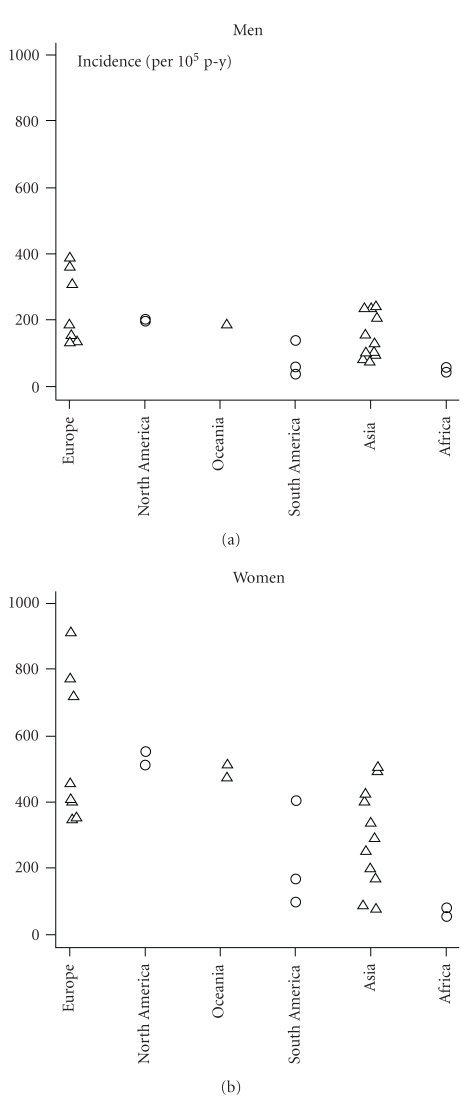
Worldwide geographic variation in hip fracture incidence.

**Table 1:** Age standardized hip fracture rates (per 100,000) across different continents.

Continent	Country	Men	Women
North America	United States, Minnesota	201.6	511.5
	United States	197.2	553.5
Europe	England	143.6	418.2
	Sweden	302.7	709.5
	Norway,	352	763.6
Oceania	New Zealand	197	516
	Australia	187.8	504.2
South America	Mexico	98	169
	Argentina	137	405
Africa	Cameroon	43.7	52.1
Asia	China, Beijing	87	97
	Iran	127.3	164.6
	Japan	99.6	368
	Kuwait	216.6	316
	Singapore	152	402
	Hong Kong	193	484.3
